# Anesthesia and Oncology: Friend or Foe?

**DOI:** 10.3389/fonc.2022.802210

**Published:** 2022-03-14

**Authors:** Bigna S. Buddeberg, Manfred D. Seeberger

**Affiliations:** ^1^ Clinic for Anesthesia, Intermediate Care, Prehospital Emergency Medicine and Pain Therapy, University Hospital Basel, Basel, Switzerland; ^2^ Medical School, University of Basel, Basel, Switzerland

**Keywords:** anesthesia, cancer, cancer recurrence, propofol, volatile anesthesia

## Abstract

Cancer is a leading cause of death, and surgery is an important treatment modality. Laboratory research and retrospective studies have raised the suspicion that the choice of anesthetics for cancer surgery might affect the course of cancerous disease. The aim of this review is to provide a critical overview of the current state of knowledge. Inhalational anesthesia with volatiles or total intravenous anesthesia (TIVA) with propofol are the two most commonly used anesthetic techniques. Most data comparing volatile anesthetics with TIVA is from either *in vitro* or retrospective studies. Although conflicting, data shows a trend towards favoring propofol. Opioids are commonly used in anesthesia. Data on potential effects of opioids on growth and recurrence of cancer are scarce and conflicting. Preclinical studies have shown that opioids stimulate cancer growth through the µ-opioid receptor. Opioids also act as immunosuppressants and, therefore, have the potential to facilitate metastatic spread. However, the finding of an adverse effect of opioids on tumor growth and cancer recurrence by some retrospective studies has not been confirmed by prospective studies. Regional anesthesia has not been found to have a beneficial effect on the outcome of surgically treated cancer patients, but prospective studies are scarce. Local anesthetics might have a beneficial effect, as observed in animal and *in vitro* studies. However, prospective clinical studies strongly question such an effect. Blood products, which may be needed during extensive cancer surgery suppress the immune system, and data strongly suggest a negative impact on cancer recurrence. The potential effects of other commonly used anesthetic agents on the outcome of cancer patients have not been sufficiently studied for drawing valid conclusions. In conclusion, laboratory data and most retrospective studies suggest a potential advantage of TIVA over inhalational anesthesia on the outcome of surgical cancer patients, but prospective, randomized studies are missing. Given the state of weak scientific evidence, TIVA may be used as the preferred type of anesthesia unless there is an individual contraindication against it. Studies on the effects of other drugs frequently used in anesthesia are limited in number and quality, and have found conflicting results.

## Introduction

According to estimates from the World Health Organization, cancer is the first or second leading cause of death in over half of the countries worldwide and is expected to take over the lead in all countries during the course of the 21st century (1). Most solid organ tumors are amenable to surgery. Sixty percent of cancer patients undergo surgical tumor resection, and 80% receive anesthesia at some point for either diagnostic, therapeutic, or palliative procedures (2–4). Despite advances in cancer treatment, cancer recurrence and metastasis remain common and lead to significant morbidity and mortality. Alarmingly, there is an increasing body of evidence that surgery and other perioperative interventions such as anesthesia create an environment conducive to the growth and spread of residual cancer cells.

For cancer recurrence to occur, two requirements need to be met. There need to be residual cancer cells that act as seeds for the recurrent cancer, and these cancer cells need to escape recognition by the host’s immune system. Seeding of tumor cells after initial surgical removal of the primary tumor can occur through four pathways (5): local recurrence from residual tumor cells at the resection site; lymph node metastasis from tumor cells released into the lymphatic system; distant organ metastasis from tumor cells released into the circulation; and seeding within a body cavity. To protect the body against tumor growth and recurrence, the body has two lines of defense: the innate immune system, which eliminates cancer cells without prior sensitization, and the adaptive immune system, which is antigen specific. The innate immune system consists primarily of myeloid cells (mononuclear and polymorphonuclear phagocytes) and to a lesser degree of natural killer (NK) cells (6). The cells of the innate immune system initiate the adaptive immune response by activating CD4+ T cells, CD8+ T cells, and B cells. Together, the innate and the adaptive immune systems fight to eliminate tumor cells. However, immune escape is common and eventually leads to cancer progression. If tumor cells survive the elimination phase by the innate and adaptive immune response, they enter the so-called equilibrium phase. In this phase, the adaptive immune response no longer manages to eliminate the tumor cells, rather they are kept in a state of dormancy. Eventually, the tumor cells manage to overcome the equilibrium phase and enter the escape phase where tumor growth occurs. In this phase, the tumor cells produce various cytokines such as vascular endothelial growth factor (VEGF) and transforming growth factor-β (TGF-β), which lead to further promotion of tumor growth (7). However, much of this knowledge is derived from preclinical studies. The processes in the human body are highly complex, and findings from preclinical studies cannot be directly translated to humans. Effects of the stress response to surgery as well as effects from therapies such as chemo- and radiotherapy and other drugs all modulate the response of the human body to cancer treatment.

After surgery, local and systemic reactions lead to an initial pro-inflammatory state, followed by a phase of immunosuppression during which the body’s ability to clear cancer cells is reduced. Locally, tumor resection causes tissue injury with a resulting inflammatory process. The inflammatory process is characterized by the release of prostaglandins, cytokines, tumor necrosis factor α (TNF-α), and chemokines. These humoral factors attract macrophages, neutrophils, and fibroblasts necessary for wound healing, but they also promote the viability and proliferation of residual cancer cells (5, 8, 9). In addition, surgery can lead to disrupted perfusion resulting in local hypoxia. Hypoxia causes the expression of hypoxia-inducible factor-1-α (HIF-1-α) and VEGF. HIF-1-α promotes tissue repair but also proliferation of residual cancer cells. VEGF causes angiogenesis as well as lymphatic dilatation, which facilitates escape of cancer cells *via* the hematologic and lymphatic pathway. It has been shown that postoperative wound complications were strongly associated with increased tumor recurrence in breast cancer patients (10). On a systemic level, surgical stress activates the hypothalamic-pituitary-adrenal axis. This activation occurs from the time of surgery until 3-4 days postoperatively. It leads to a secretion of ACTH and cortisol, which increases the production of glucocorticoids, catecholamines, and cytokines and results in immunosuppression (8, 9). In addition, the activation of the sympathetic nervous system causes an immunosuppressive effect *via* sympathetically-innervated lymphoid organs (9).

The aim of this narrative review is to provide a critical overview of the current state of knowledge of the effects of commonly used anesthetic agents on cancer growth and patient survival.

## Volatile Anesthetics

It is increasingly recognized that volatile anesthetics have an effect not only on the central nervous system, but also on other organ systems including the immune system. Volatile anesthetics modulate the innate as well as the adaptive immune response (11, 12). They suppress innate immunity mainly through suppression of neutrophils, dendritic cells, NK cells, and resident tissue macrophages. The adaptive immune system is suppressed by a decrease in proliferation of lymphocytes and an increase in lymphocyte apoptosis. In addition to its effects on the innate and adaptive immune system, volatile anesthetics also affect the immune system indirectly through their impact on stress hormone levels. Surgery leads to the stimulation of the hypothalamic-pituitary-adrenal axis and to the release of glucocorticoids and catecholamines, which can alter the immune response systemically. This effect was found to be more pronounced after anesthesia with volatile versus intravenous agents (13). The suppression of the immune system by volatile anesthetics can be beneficial in cases of sterile inflammation such as ischemia-reperfusion, but in cancer surgery it has the potential to promote tumor recurrence and metastasis (11).

Numerous *in vitro* studies have investigated the effect of volatile anesthetics on human cancer cell lines. Benzonana et al. exposed renal cell carcinoma cells for 2h to different clinical concentrations of the volatile anesthetic isoflurane (0.5-2%) (14). They measured levels of VEGF and hypoxia-inducible factors (HIFs), high levels of which are associated with poor prognosis. In addition, they looked at cell migration. Cultures exposed to isoflurane showed higher levels of HIFs and VEGFs, they contained more cells, more actively proliferating cells, and the cells exhibited greater migration. Iwasaki et al. exposed human ovarian carcinoma cells to high levels of isoflurane (3%), sevoflurane (3.6%), or desflurane (10.3%) for 2h and studied metastasis related gene expression profiles (15). All three volatile anesthetics altered expression of 70 out of 81 metastasis-related genes. Desflurane had the greatest effect, followed by sevoflurane and isoflurane. Luo et al. also studied the effect of isoflurane on ovarian cancer cells (16). They exposed the cells for 2h to 2% isoflurane and studied the expression of markers involved in cell proliferation, angiogenesis, and migration. Isoflurane exposure increased the expression of insulin-like growth factors, VEGFs, and angiopoietin. Cell cycle progression and cell proliferation were also increased. Ciechanowicz et al. exposed non-small cell lung adenocarcinoma (NSCLC) cells and renal cell carcinoma cells to 3.6% sevoflurane for 2h (17). In NSCLC cells, sevoflurane reduced cell viability and enhanced chemosensitivity to cisplatin, but had no effect on cell migration. In renal carcinoma cells, however, sevoflurane enhanced cell viability, chemoresistance to cisplatin, and cell migration.

Taken together, most *in vitro* studies have found that exposure of cancer cell lines to volatile anesthetics reduced apoptosis of the cancer cells and favored their proliferation, migration, and chemoresistance.

Clinical studies often compare volatile anesthesia to total intravenous anesthesia with propofol. These studies are discussed in the comparative chapter following the next section on propofol.

## Propofol

*In vitro* cancer cell studies have found that propofol may have specific effects on cancer cell apoptosis and proliferation. Propofol exerts an anti-tumor effect mainly but not exclusively by a down-regulation of matrix metalloproteinases (MMPs) (18). This downregulation leads to a decrease in cancer cell proliferation and invasion and an increase in cancer cell apoptosis. Such an effect has been shown for *in vitro* cell cultures of pancreatic cancer cells (19), ovarian cancer cells (20, 21), hepatocellular carcinoma cells (22, 23), gastric cancer cells (24), glioma cells (25), osteosarcoma cells (26), lung adenocarcinoma cells (27), colon carcinoma cells (28) and breast cancer cells (28). Besides downregulating MMPs, propofol has been found to also exert an anti-tumor effect by other pathways. In non-small-cell lung cancer cells, propofol reduced the aggressiveness of cancer cells by reducing the upregulation of HIF-1α (29). In esophageal cancer cell cultures, propofol reduced proliferation, invasion, and angiogenesis by reducing extracellular signal-regulated kinases, which lead to a reduced expression of VEGF and MMP-9 (29). In osteosarcoma cells, propofol decreased sarcoma cell proliferation and invasion, and increased apoptosis by downregulating transforming growth factor β-1 (TGF-β-1), an immunosuppressing cytokine (30). In breast cancer cells, propofol reduced migration by reducing neuroepithelial cell transforming gene 1 (NET1), a gene associated with promoting migration in adenocarcinoma cells (31).

However, propofol has not only been associated with potentially beneficial anti-tumor effects in tumor cell studies. In a breast cancer cell model, Garib et al. found an increase in the percentage of migrating cells after exposing breast cancer cells to propofol (32). The same group also reported that propofol increased the migration of breast cancer cells *via* the activation of the γ-aminobutyric acid-A (GABA-A) receptor (33). Similarly, Meng et al. observed an increase in proliferation and migration in a human breast cancer cell lines after treatment with propofol (34).

Cancer cell studies have found that propofol may also alter the sensitivity of cancer cells to chemotherapeutic agents. Chen et al. reported that propofol enhances paclitaxel-induced apoptosis in ovarian cancer cells through the suppression of the transcription factor slug (35). In pancreatic cancer cells, propofol has been shown to increase gemcitabine sensitivity by inhibition of NF-kB activity (36), and in cervical cancer cells propofol enhanced cisplatin-induced apoptosis (37). In glioma cells, however, propofol was found to depress cisplatin cytotoxicity by reducing gap junctions between the cells (38).

Animal studies provide additional information on the effects of propofol on tumor growth. Cui et al. injected lung cancer cells into the axilla of mice, and once the tumor had reached a size of 3-5 mm, the mice were divided into three groups (39): one receiving no treatment, one receiving saline, and one receiving propofol. After 30 days, mice in the propofol group had significantly decreased tumor size and weight. Kushida et al. injected thymoma cells subcutaneously into mice (40). The cytotoxic activity of T cells collected from the spleen was then measured. The cytotoxicity of the T cells was significantly greater in mice treated with propofol than those treated with intralipid or saline. Further, tumor growth was significantly suppressed.

Taken together, most *in vitro* and animal studies suggest a beneficial effect of propofol on cancer cell apoptosis and proliferation. Few cancer cell studies have also reported that propofol might alter the sensitivity of cancer cells to chemotherapeutic agents.

## Volatile Anesthesia Versus Total Intravenous Anesthesia

The effects of volatile versus intravenous anesthetics have been studied in some *in vitro* and animal studies comparing their effects on cancer cell growth, and in a large number of retrospective analyses on the outcome of cancer patients.

*In vitro*, Huang et al. exposed prostate cancer cell lines either to isoflurane or to propofol and assessed the malignant potential by evaluating expression levels of HIFs and the downstream effects (41). Isoflurane induced an upregulation of HIFs and, thus, an increase in proliferation, migration, and chemoresistance of cancer cells. In contrast, propofol inhibited expression of HIFs. Jaura et al. collected serum from women who had undergone surgery for breast cancer either with propofol anesthesia combined with a paravertebral block or with sevoflurane anesthesia in combination with opioids (42). Estrogen receptor negative breast cancer cells were exposed to serum from either the propofol-paravertebral block group or to serum from the sevoflurane-opioid group. Apoptosis of the cancer cells was significantly reduced in the cell culture exposed to the serum from the sevoflurane-opioid group. In another *in vitro* study with serum from women undergoing breast cancer surgery, Buckley et al. compared effects of serum from a propofol-paravertebral block group and a sevoflurane-opioid group on human NK cell cultures (43). The serum of women from the propofol-paravertebral block group led to a greater cytotoxicity of the NK cells than the serum of women from the sevoflurane-opioid group. Lim et al. studied breast cancer cells co-cultured with NK cells and cytotoxic T lymphocytes (CTL) (44). They exposed these cultures to serum collected from women who had undergone breast cancer surgery either with propofol-based anesthesia or sevoflurane-based anesthesia. In contrast to the other laboratory studies, propofol was not superior to sevoflurane in this study, as no difference in NK cell count, CTL count, or apoptosis rate was detected between the groups. In an animal study, Melamed et al. injected breast cancer cells into rats and anesthetized the animals for one hour with ketamine, thiopental, halothane, or propofol (45). All anesthetics except propofol significantly reduced NK cell activity and increased lung tumor retention and the occurrence of lung metastasis.

A large number of retrospective clinical studies compare the effects of volatile versus intravenous anesthesia on the outcome of cancer patients. Better overall survival after anesthesia with propofol compared to anesthesia with volatile agents has been reported for gastric cancer (46), colon cancer (47), breast cancer (48), esophageal cancer (49), and hepatocellular carcinoma (50). Wigmore et al. retrospectively analyzed patients with different types of solid organ cancers undergoing resective surgery and found that mortality was approximately 50% higher with volatile anesthesia than with intravenous anesthesia (51). Lee et al. found no difference in overall survival after propofol anesthesia compared to sevoflurane anesthesia in patients undergoing mastectomy for breast cancer, but they found a lower recurrence rate after propofol anesthesia (52). Similarly, Hasselager et al. found a weak association between colorectal cancer recurrence and exposure to inhalational anesthesia when compared to total intravenous anesthesia, but no association between all-cause mortality or disease-free survival (53). Jun et al. similarly found worse recurrence-free survival after anesthesia with volatile agents compared to propofol in patients with esophageal cancer (49). In contrast, no difference in overall patient survival and recurrence-free survival between total intravenous anesthesia and volatile anesthesia was reported for non-small cell lung cancer (54) and breast cancer (55, 56). Enlund et al. looked at patients after radical cancer surgery for breast, colon, or rectal cancer and compared 1- and 5-year overall survival rates between propofol and sevoflurane anesthesia. They found an apparent advantage of propofol over sevoflurane, which disappeared after adjustment for several confounders (57).

One of the very few prospective, randomized trials was published by Oh et al. who studied whether propofol is less immunosuppressive than sevoflurane (58). Indicators of a potential immunosuppressive effect of the anesthetics analyzed in this study were clusters of differentiation 39 and 73. These clusters are expressed on the surface of regulatory T cells that promote cancer recurrence and metastasis by suppressing immune cells. In blood samples collected from women undergoing breast cancer surgery who were randomized to either propofol or sevoflurane anesthesia, changes in cluster differentiation 39 and 73 expression did not differ between the two groups (58). In line with these findings are the conclusions drawn by a recent meta-analysis of 23 randomized controlled trials examining perioperative inflammation after general anesthesia using propofol compared to sevoflurane (59). The authors of the meta-analysis found an increase in the mean inflammatory biomarker levels of IL-6, IL-10, TNF-α, and C-reactive protein (CRP) after surgery but no difference between propofol and sevoflurane.

Finally, a meta-analysis from 2019 included ten studies to compare the potential effects of intravenous versus volatile anesthesia on recurrence-free survival and overall survival in cancer patients (60). Based on six of these studies, the meta-analysis came to the conclusion that the use of TIVA was associated with improved recurrence-free survival in breast, esophageal, and non-small-cell lung cancer. Further, based on eight studies with a total of 18,778 patients, the meta-analysis found that overall survival was also improved with the use of TIVA. It must be noted, however, that nine of the ten studies were retrospective in design, and that the prospective study was much too small to reliably analyze patient outcomes. The authors conclude that their findings suggest a beneficial effect of propofol-based anesthesia on cancer outcomes but indicate the need for prospective studies before reliable conclusions can be drawn.

Taken together, laboratory studies and most retrospective studies suggest a potential beneficial effect of propofol-based TIVA on the outcome of cancer patients undergoing surgery. Data from randomized-controlled trials is scarce and did not confirm the beneficial effects of propofol seen in laboratory and retrospective studies. Results of further ongoing prospective, randomized studies are needed before final conclusions can be drawn. Meanwhile, TIVA may be used as the preferred type of anesthesia in patients with cancerous disease unless there is an individual contraindication against it.

## Opioids

Pain and stress have been shown to favor cancer dissemination in rodents (61). Therefore, any drug used to treat pain has the potential to alter this response. Opioids are used widely in cancer patients to treat perioperative and cancer-related pain in the palliative setting. Research regarding the role of opioids in cancer dissemination is conflicting, and there is evidence that not all opioids exert the same effect on the immune system. Morphine seems to have positive and negative effects on the immune system. Fentanyl and codeine seem to have mainly immunosuppressive effects. Tramadol has mainly immunostimulating effects. Buprenorphine, oxycodone, and hydromorphone appear to be neutral (7). It is believed that opioids exert their influence on tumor growth and progression mainly through activation of the µ-opioid receptor. This belief is strengthened by the observation that patients with advanced cancer who were treated with the µ-opioid-receptor antagonist methylnaltrexone had higher disease-free survival (62). Reduced cancer cell growth in lung carcinoma cells after treatment with methylnaltrexone was also seen *in vitro* (63). It further could be shown that naloxone inhibited cell proliferation and increased cell death in human estrogen-receptor negative breast cancer cells *in vitro* and lead to reduced cancer growth in mice (64).

*In vitro* and mouse studies found that morphine decreases tumor growth in breast (65), colon (66), and melanoma (67) cancer cells. Morphine was also shown to decrease transendothelial migration of leukocytes and reduce angiogenesis in lung cancer cells (68). In a mouse model of breast cancer, morphine lead to a reduction in tumor growth and to a reduction in circulating levels of MMP-9 and urokinase-like plasminogen activator (69). Harimaya et al. observed that morphine also reduced the adhesion, invasion, and metastasis of colon cancer cells *in vitro* by the regulation of MMPs (66). In contrast, morphine increased tumor growth in breast (70), sarcoma, and leukemia (71) cancer cells *in vitro* and in mouse models. In breast (72) and lung cancer (73), it promoted invasion and migration of cancer cells *via* the upregulation of MMPs and in colon cancer *via* the upregulation of urokinase plasminogen activator (74). A meta-analysis of experimental animal studies on the effect of treatment with analgesics was published in 2015. The authors came to the conclusion that there is no evidence that treatment with any analgesics including opioids increases the occurrence of metastases (75).

Clinical studies have also failed to provide clear evidence on potential effects of opioids on tumor growth and cancer recurrence. One reason is that most studies are retrospective in design, which limits the reliability of their findings. In addition, drawing conclusions from several studies performed in patients with the same type of cancer is limited by the large heterogeneity of the studies. Furthermore, many studies compared general anesthesia combined with regional anesthesia to general anesthesia combined with opioids. It is, therefore, not clear if observed effects, if any, are due to regional anesthesia or to opioids.

There is one large prospective Danish cohort study of more than 34,000 women with newly diagnosed breast cancer (76). After a mean follow-up of more than 8 years, the authors failed to detect any correlation between opioid prescription and breast cancer recurrence, regardless of opioid type, chronicity of use, or cumulative dose. This finding is in agreement with those of two recently published prospective trials comparing general anesthesia with regional analgesia versus general anesthesia plus opioids (77, 78). Sessler et al. compared patients who underwent breast cancer resection and who received general anesthesia either with propofol plus a paravertebral block or with sevoflurane and postoperative opioids. Recurrence of cancer was similar in the two patient groups during a median follow-up period of 36 months (77). In the second prospective study in patients with colorectal cancer, disease-free survival after 5 years was not affected by the use of thoracic epidural analgesia vs. patient-controlled opioid analgesia at the time of surgery (78). There is also a recent systematic review of published data on the effect of perioperative opioids on colorectal cancer recurrence. However, the authors were unable to perform a quantitative analysis because of the great heterogeneity of the studies (79).

Finally, there are many retrospective studies focusing on different types of cancer with quite conflicting results. Differences between these studies include study design and specific focus, size, quality, and complexity of data. One retrospective analysis of almost 500 patients with stage IV prostate cancer found that higher opioid requirements were associated with shorter progression-free and overall survival (80). However, effects of tumor volume on pain and, consequently, opioid use are potential confounders of this finding as the authors of the study indicate in the limitations section. Another retrospective study of 901 patients with NSCLC analyzed the association between intraoperative fentanyl dose and cancer recurrence (81). In stage I patients, the authors found a trend towards increased risk for recurrence and decreased overall survival with higher fentanyl doses. However, no effect was found in stage II and III patients. In patients with lung adenocarcinoma stage I to III, another retrospective study reported an association between intraoperative opioid exposure and worse overall survival (82). Another retrospective study by Biki et al. in patients who had undergone open radical prostatectomy reported an estimated 57% lower risk of cancer recurrence in patients who had received general anesthesia with epidural analgesia compared to patients with general anesthesia and postoperative opioid analgesia (83).

Taken together, the few prospective studies all failed to detect a negative effect of perioperatively-administered opioids on tumor growth and cancer recurrence. This fact questions the findings of previous retrospective studies. Confounding effects (e.g. of tumor volume on pain and thus opioid use) might be reasons for the association between opioid use and outcome found in those earlier studies. The current state of knowledge based on prospective studies strongly suggests to continue using opioids as strong analgesics in cancer patients who frequently suffer from intense pain. Nevertheless, further prospective studies are needed to definitively clarify potential effects of opioids on growth and recurrence of different types of malignant tumors.

## Regional Anesthesia

Regional anesthesia, used either alone or in combination with general anesthesia reduces the perioperative stress response, perioperative pain, and therefore, the perioperative opioid requirements as well. Whether regional anesthesia has a direct influence on cancer recurrence is less clear.

*In vitro* studies of serum from patients who underwent cancer surgery showed a potential benefit of regional anesthesia. Xu et al. collected serum from patients undergoing colon cancer surgery with general anesthesia either by propofol and epidural analgesia or by sevoflurane and opioid analgesia (84). *In vitro*, serum from patients of the propofol-epidural group showed inhibited proliferation and invasion of colon cancer cells and induced apoptosis more often than serum from patients of the sevoflurane-opioid group. The previously mentioned *in vitro* studies of Buckley (43) and Jaura (42) showed similar beneficial effects.

Clinical data show controversial results. Prospective studies in patients with colorectal (85), abdominal (86), breast (87), and prostate (88) cancer found no beneficial effect of regional anesthesia. A Cochrane database systematic review published in 2014 analyzed whether regional anesthesia influences long-term prognosis for individuals with malignant tumors (89). The authors searched for controlled trials on general anesthesia alone versus general anesthesia combined with epidural analgesia in cancer patients. They identified four secondary analyses of controlled, prospective randomized trials with a total of 746 patients with abdominal (two studies), colon, and prostate cancer. The systematic review revealed no difference between the groups without vs. with additional epidural analgesia, and the authors concluded that evidence for the benefit of regional anesthesia techniques on tumor recurrence is inadequate. Similarly, another meta-analysis from 2017, which included 28 studies with an array of cancers also looked at the potential benefit of regional anesthesia (90). This meta-analysis also found no benefit of regional anesthesia on overall survival, recurrence-free survival, or biochemical recurrence-free survival. Finally, a number of retrospective studies in patients with colon (91), abdominal (92), and breast (93) cancer also found no beneficial effect of regional anesthesia. In contrast, a meta-analysis of 21 studies published in 2016 found that the use of neuraxial anesthesia was associated with improved overall survival in patients undergoing cancer surgery, particularly in those with colorectal cancer (94). It also reported a potential association between neuraxial anesthesia and reduced risk of cancer recurrence. It must be noted, however, that only 5 of the 21 studies were prospective trials, and that only one of them found an association between neuraxial anesthesia and improved survival. The retrospective study by Biki et al., which also found an association between epidural anesthesia and reduced cancer recurrence, has already been mentioned (83).

Taken together, there is no adequate scientific evidence for a beneficial effect of regional anesthesia on the outcome of surgically treated cancer patients. Retrospective studies have found conflicting results, and nearly all prospective studies have failed to detect any beneficial effect of regional anesthesia. Therefore, complementing general anesthesia with regional techniques may be reasonable for optimizing patient comfort, but it does not seem to improve patient outcome.

## Local Anesthetics

The effect of local anesthetics on tumor growth has been studied in several *in vitro* and animal studies, but clinical studies are missing. The clinical administration of intravenous lidocaine during anesthesia has been promoted by the observation that it is associated with a lower use of opioids, a lower incidence of nausea and vomiting, and faster recovery from postoperative ileus (95–97). Furthermore, lidocaine has potent anti-inflammatory activity *via* the modulation of IL-6, IL-8, leukotrienes, and polymorphonuclear leukocytes (9).

Several *in vitro* studies and trials in mice have demonstrated the anti-cancer potential of lidocaine and other local anesthetics. After incubating two breast cancer cell lines with high concentrations of lidocaine, bupivacaine, and four other local anesthetics, Li et al. observed significantly inhibited cell viability and induced cytotoxicity (98). At concentrations reached by regional anesthesia, however, none of the local anesthetics affected cell viability or migration in the included patients. Xuan et al. exposed ovarian and prostate carcinoma cells *in vitro* with bupivacaine at clinically relevant concentrations and observed reduced cell viability and inhibited cellular proliferation in both cell lines (99). Another *in vitro* study found that the growth of human hepatocellular carcinoma cells was inhibited in a dose- and time-dependent manner by lidocaine (100). When human hepatocellular carcinoma cells were transferred into mice, intraperitoneal injection of lidocaine markedly suppressed tumor growth. Chamaraux-Tran et al. exposed normal breast epithelial cells and three tumor breast epithelial cell lines to clinically relevant concentrations of lidocaine and investigated cell viability and migration (101). Lidocaine reduced the viability of all three malignant cell lines and inhibited migration but had no effect on the normal breast epithelial cells. When they injected breast cancer cells intraperitoneally into mice, addition of intraperitoneal lidocaine improved survival of the mice. Also in a murine breast cancer model, other investigators found that addition of lidocaine during anesthesia with sevoflurane for tumor resection reduced cancer progress with pulmonary metastasis but had no effect when ketamine and xylazine had been used for anesthesia (102).

Taken together, some *in vitro* and animal studies suggest a potential beneficial, possibly dose-dependent, effect of local anesthetics on tumor growth and metastatic disease. The prospective clinical studies reported in the section on regional anesthesia strongly question such an effect at plasma levels induced by epidural anesthesia. Whether potentially higher plasma levels of lidocaine, when perioperatively infused as a component of multimodal analgesia (103), have an effect on outcome of cancer patients needs to be investigated in prospective trials.

## Blood Transfusion

Cancer surgery can be extensive, and therefore, blood transfusion can be lifesaving. However, transfusion of allogenic blood involves specific immunologic risks. Even after leucocyte reduction, the few remaining leucocytes in packed red blood cells (pRBC) have the ability to modulate the immune response of the recipient. In addition to residual leucocytes, there are also biologically active cytokines, non-polar lipids, and a mixture of pro-inflammatory lysophosphatidylcholines in pRBC. Lysophosphatidylcholines activate NK cells, T lymphocytes, and dendritic cells and stimulate the production of pro-inflammatory cytokines. The overall effect of these biological substances is immunosuppression and tumor-promotion (104). Atzil et al. studied the effect of blood transfusion on cancer progression in a mammary adenocarcinoma and a leukemia rat model (105). Blood transfusion was found to be an independent and significant risk factor for tumor progression in both models, regardless whether allogenic or autogenic blood was used. Duration of blood storage was the critical determinant of this effect and, surprisingly, aged erythrocytes rather than leukocytes mediated it. Hod et al. could demonstrate in a murine model that the transfusion of stored red blood cells increased plasma non-transferrin-bound iron, increased acute tissue iron deposition, and initiated inflammation (106).

Available data from clinical studies has been summarized in several meta-analyses. A Cochrane review from 2006 including 36 studies with more than 12,000 patients analyzed the role of perioperative blood transfusion on colorectal cancer recurrence (107). The effect of perioperative blood transfusion on cancer recurrence yielded an odds ratio (OR) of 1.42 (95% CI: 1.2 to 1.67) against transfused patients. The fact that 26 of the 36 studies in this Cochrane review had been performed retrospectively might question the validity of this finding. However, a separate analysis of the studies with higher quality, and of the ten prospective studies yielded similarly significant ORs. Li et al. performed a meta-analysis to look at the association between allogenic or autologous blood transfusion and survival in patients after radical prostatectomy (108). Data from 26,000 patients in ten studies was included. They found that allogenic blood transfusion was significantly associated with worse recurrence-free survival, overall survival, and cancer-specific survival. In patients with autologous blood transfusion, this effect was not seen. Agnes et al. did a meta-analysis on the association between allogenic perioperative blood transfusion and recurrence of cancer in patients who had undergone curative gastrectomy for gastric cancer (109). Perioperative blood transfusion was associated with worse overall survival, disease-free survival, and disease-specific survival and an increased number of postoperative complications. Similar findings for bladder cancer were found in a meta-analysis by Cata et al. (110). It must be noted, however, that the meta-analyses by Li, Agnes, and Cata all rely on retrospective studies, which require cautious interpretation of the results.

Taken together, there is meta-analytic evidence of an association between allogenic blood transfusion and increased number of postoperative complications, cancer recurrence, and worse patient survival. These findings fit to laboratory evidence of immunosuppression induced by transfused blood. Therefore, using a restrictive transfusion threshold in cancer patients and in general is mandatory, although more aggressive forms of cancer may have contributed to worse outcome in transfused patients by necessitating more blood transfusions.

## Non-Steroidal Anti-Inflammatory Drugs

Cyclooxygenase-2 (COX-2) is a key enzyme in the synthesis of prostaglandins (PG) and has been demonstrated to play an important role in the development, growth, and invasion of several cancer types (111, 112). NSAIDs, selective COX-2-inhibitors, and aspirin, thus, have a potential role in the treatment and prevention of malignant tumors through different pathways. Kashiwagi et al. demonstrated that aspirin downregulates androgen receptors and prostate-specific antigens in prostate cancer cells *in vitro* (113). They also found that aspirin upregulates the prostaglandin receptor EP3. Activation of EP3 receptors leads to a decrease in androgen receptors. Aspirin and EP3 receptor agonists, therefore, have the potential to modulate prostate cancer growth. Evidence for the role of COX-2 and PGs in the development of colorectal cancer could be gained from a murine model of adenomatous polyposis (114). In a mouse model of hepatocarcinoma, the selective COX-2-inhibitor celecoxib delayed growth of the tumor (115).

A Finnish population-based study revealed NSAID use to be associated with an increased risk of prostate cancer, while aspirin use was associated with a decreased risk (116). Another database study looked at prostate cancer patients treated with either surgery or radiotherapy (117). They compared prostate cancer-specific mortality between patients receiving anticoagulant treatment and patients with no anticoagulant treatment and found that prostate cancer-specific mortality was lower in both patients treated with surgery and radiotherapy if they received anticoagulation treatment. A subgroup analysis revealed that the reduced mortality in patients receiving anticoagulation was mainly due to the effect of aspirin. Evidence from a prospective observational study in humans found that the chronic use of NSAIDs reduced the risk of developing gastrointestinal cancer (118). Moreover, the largest body of evidence supports the beneficial effects of NSAIDs in colorectal cancer prevention and adjuvant treatment (119). The adenoma prevention with celecoxib trial examined the efficacy and safety of celecoxib for colorectal adenoma prevention in patients with an increased risk of colorectal cancer. It found that celecoxib had a long-term protective effect on the prevention of colorectal adenoma progression but increased the risk of renal and hypertensive events and cardiac disorders (120, 121). In line with this, Ng et al. found in a prospective observational study that aspirin and COX-2 inhibitors may be associated with improved outcomes in stage III colon cancer (122). Overall, it can be said that the use of NSAIDs and COX-2 inhibitors is associated with a reduced risk of occurrence and progression of colorectal cancer (119). This beneficial effect, however, is offset by the increased risk of cardiovascular events and gastrointestinal toxicity. The risk-benefit analysis, therefore, prevents their widespread use in prevention and adjuvant use in colorectal cancer. Among NSAIDs, aspirin in low doses is the only agent with a potential overall benefit in chemoprevention and adjuvant therapy in colorectal cancer due to its protective nature against cardiovascular events and its low gastrointestinal toxicity. In breast cancer, however, a Danish registry study indicated that post-diagnostic use of aspirin, NSAIDs, or selective COX-2-inhibitors was not associated with a reduced rate of recurrence. However, pre-diagnostic use was associated with a reduced rate of recurrence (123). In contrast to these findings, Huang et al. found in a meta-analysis of 16 studies published in 2015 that NSAID and aspirin use after, but not before diagnosis was associated with improved breast cancer survival including breast cancer-specific mortality, all-cause mortality, relapse, and metastasis (124). These conflicting findings clearly indicate the lack of reliable evidence in the absence of adequate prospective trials.

When it comes to perioperative administration of NSAIDs and cancer recurrence, data is even more sparse. In breast cancer, retrospective data showed an association between the intraoperative administration of ketorolac and reduced recurrence rate in patients undergoing surgery (125–127). In patients with ovarian cancer, a retrospective study found the perioperative use of ketorolac to be associated with a decreased cancer-specific mortality six years after surgery. In patients with prostate cancer undergoing retropubic prostatectomies and NSCLC, there was no association between perioperative NSAIDs and cancer recurrence (128, 129).

To summarize the existing evidence on the effect of NSAIDs on cancer recurrence, we would like to mention a systematic review from 2017 (130). The authors found 16 trials but did not perform a meta-analysis because of the high heterogeneity and low quality of the available studies. This fact clearly indicates that we lack adequate scientific evidence to make any recommendation on the use of NSAIDs, aspirin, and COX-2-inhibitors based on outcome aspects in cancer patients.

## Ketamine

There has been a revival in the use of ketamine in recent years. Ketamine is a NMDA-receptor antagonist and has agonistic properties on the µ- and δ-opioid receptors. It has a strong analgesic effect and, therefore, can reduce the amount of preoperatively-used opioids (9). Its effect on the immune system and cancer recurrence is less clear.

The murine study by Melamed et al. has already been mentioned (45). They anesthetized rats with either thiopental, ketamine, halothane, or propofol for one hour and then injected them with breast cancer cells. All anesthetics except propofol decreased NK cell activity and increased cancer cell retention and lung metastasis. Ketamine increased lung metastasis most potently (45). Forget et al. looked at the influence of ketamine on NK cell activity and occurrence of lung metastasis in rats injected with breast cancer cells (131). Ketamine only led to a reduction of NK cell activity in unoperated rats. However, ketamine reduced the number of metastasis in operated animals, a finding that is in full contrast to the study by Melamed et al. (45).

Clinical studies are scarce and partially conflicting. In a prospective and randomized study of patients with colorectal cancer, Cho et al. found that the administration of intraoperative low-dose ketamine did not have a favorable impact on overall postoperative NK cell activity, inflammatory response, and prognosis (132). In a small randomized trial in patients undergoing minimally invasive prostatectomy for prostate cancer, Kawaguchi et al. also failed to detect an immunomodulatory effect by ketamine (131). In a retrospective study, Forget et al. also found no beneficial effect of ketamine on cancer recurrence in patients who had undergone mastectomy for breast cancer (127). In another retrospective study, Connolly et al. made the contrary finding of improved recurrence-specific survival in patients with early-stage lung adenocarcinoma who had received ketamine intraoperatively (82).

In summary, there is no adequate scientific evidence to advocate the perioperative use of ketamine for improving outcome in cancer patients.

## α2-Agonists

The centrally acting α2-agonists clonidine and dexmedetomindine reduce the release of noradrenaline and, therefore, dampen the sympathetic stress response. They have a sedative effect and are used perioperatively to reduce the need for opioids. There is little data available on their effect on the immune system and cancer recurrence.

Cheng et al. could demonstrate *in vitro* that dexmedetomidine inhibited the maturation of dendritic cells, which are important players in the immune response (133). Wang et al. demonstrated *in vitro* that dexmedetomidine promoted cell proliferation and migration and upregulated anti-apoptotic proteins in human lung carcinoma cells and human neuroglioma cells (134). In a murine model of lung carcinoma, dexmedetomidine did not alter tumor growth (134). In contrast, dexmedetomidine and clonidine increased tumor growth and metastasis in murine breast cancer models (135, 136). Other investigators found that dexmedetomidine increased tumor cell retention and growth of metastases in different animal models of breast, lung, and colon carcinoma (137), and that it had a tumor promoting effect through the increasing production of VEGF in a murine lung cancer model (138). In surgical lung cancer patients, these investigators found that dexmedetomidine induced the proliferation of M-MDSC cells, which have a potent proangiogenic ability (138).

Prospective outcome studies in patients have not been published, and retrospective studies are scarce and inconsistent. In lung cancer patients undergoing surgery, Connolly et al. did not find an association between the administration of dexmedetomindine and overall survival and recurrence-specific survival (82), while Cata et al. found that dexmedetomidine was associated with reduced overall survival but not with recurrence-free survival (139).

Taken together, the effects of α2-agonists on the outcome of cancer patients, if any, are unknown.

## Steroids

Steroids inhibit cyclooxygenase and lipoxygenase pathways and, thereby, reduce postoperative pain. Further, they have an antiemetic effect. For these reasons, steroids are often administered perioperatively to cancer patients. While they are part of the treatment in hematologic malignancies, their role in solid organ tumors is less clear.

In a xenograft mouse model of prostate cancer, dexamethasone led to a decrease in tumor growth and microvessel density through the downregulation of VEGF and IL-8 (140). As this effect was not seen *in vitro*, the investigators hypothesized that dexamethasone might decrease tumor growth by inhibition of tumor-associated angiogenesis. In a xenograft model of prostate cancer, Nishimura et al. also found that dexamethasone inhibited the NF-kB and IL-6 pathway and lead to reduced cancer growth (141). *In vitro* as well as in a xenograft model, Arai et al. also observed that dexamethasone inhibited growth of renal cancer cells through the inhibition of the NF-kappa-B pathway and its downstream products IL-6, IL-8, and VEGF (142). In contrast to these findings, dexamethasone mediated tumor progression in pancreatic cancer cell lines and in a pancreatic cancer cell xenograft model (143).

In one published outcome study in patients with colon cancer, based on the follow up of a previous randomized trial (144), the authors found that preoperative dexamethasone was associated with a higher rate of distant metastases five years after colectomy. However, they point out that the very small sample size (20 vs. 23 in the dexamethasone vs. placebo group, respectively) prohibits reliable conclusions. Another observational study by McSorley et al. in patients undergoing either open or laparoscopic surgery for colonic cancer looked at the effect of surgical approach and intraoperatively administered dexamethasone (145). They found a significant trend towards a lower postoperative systemic inflammatory response with the use of laparoscopic surgery and higher doses of dexamethasone. The combination of laparoscopic surgery and higher doses of dexamethasone was also associated with fewer postoperative complications. The authors also found that the use of dexamethasone was not significantly associated with either improved or poorer cancer-specific or overall survival. In another retrospective study, the same group found that preoperatively administered dexamethasone in patients undergoing surgery for colorectal cancer was associated with a lower postoperative systemic inflammatory response as evidenced by a lower CRP level (146). A systematic review and meta-analysis in patients undergoing surgery for gastrointestinal cancer found that preoperatively administered corticosteroids were associated with a reduced postoperative systemic inflammatory response and fewer postoperative complications (147). In a cohort study, patients were followed up for 5-10 years after breast cancer surgery (148). A single dose of perioperatively administered dexamethasone was not associated with increased recurrence or mortality after curative breast cancer surgery. Finally, retrospective analysis of data from a prospectively maintained database of patients undergoing pancreaticoduodenectomies for pancreatic cancer revealed that intraoperatively administered dexamethasone did not increase morbidity, was associated with a decrease in infectious complications, and an increase in overall survival (149).

More data from prospective human studies is necessary before valid conclusions on the effects of perioperatively administered steroids on the outcome of cancer patients can be made.

## Discussion

Numerous laboratory, animal, and clinical retrospective studies have investigated the impact of commonly used anesthetic agents on cancer outcome. Good high quality prospective randomized trials, however, are scarce.

Based on this insufficient scientific evidence, no firm conclusions can be drawn and no sound recommendations be made at this juncture. Findings from *in vitro* and animal studies must not be extrapolated to cancer patients undergoing surgery. Reasons are that there are multiple differences between the complex clinical situation in surgical cancer patients and the situation in artefactual cell culture studies or animal studies. Highly cultured tumor cell lines with optimal cell culture conditions are artefactual and have only limited relevance to the much more complex *in vivo* situation. It is also unclear if or to what degree anesthetic dosage and duration of exposure to such agents as used in cancer cell line studies are representative of the *in vivo* situation.

Well-controlled animal studies also differ in multiple aspects from the much more complex situation of surgical cancer patients, and it is unclear to what degree their findings can be extrapolated to humans. Differences include effects of the stress response to surgery, interaction with other drugs, or the effects of potential chemotherapy and radiotherapy in patients, conditions that are generally absent in animal models. Fever or cold are two of many additional factors that may differ between animal studies and the clinical situation in surgical patients. Fever stimulates the innate as well as the adaptive immune system. Pyrogenic cytokines produced during the induction of fever also activate the immune system (150). Cold stress, however, leads to an increased release of norepinephrine and has been associated with accelerated tumor growth in murine models, suppression of endogenous immune responses, and therapeutic resistance of tumors (150, 151). Laboratory mice are generally kept at sub-thermoneutral housing temperatures. This characteristic might potentially lead to biased outcomes in murine cancer models (151). Xenograft studies have the specific limitation that they are performed in mice with immunocompromised immune systems, which again questions their generalizability to humans.

Finally, retrospective clinical studies have inherent limitations, which limit the validity of their findings (152).

Taken together, scientific evidence is quite limited. The fundamental differences between laboratory conditions and the clinical situation as well as the limitations of retrospective clinical studies must be considered when their meaningfulness for clinical decisions in cancer patients is appraised.

Laboratory, animal, and retrospective clinical studies suggest a potential advantage of propofol-based total intravenous anesthesia over inhalational anesthesia. The few prospective clinical trials available, however, have failed to prove a benefit of propofol. In addition, the few prospective studies available have often compared propofol plus regional anesthesia with inhalational anesthesia plus opioids, making it even more difficult to isolate the effects of propofol and inhalational anesthesia. Until large prospective clinical trials are available, it is certainly not wrong to favor propofol over volatiles for maintenance of anesthesia during cancer surgery.

With regard to opioids, the evidence is conflicting. There are retrospective studies that have found higher perioperatively administered opioid doses to be associated with worse cancer outcome. However, the few available prospective studies have failed to detect a negative effect of perioperatively administered opioids on tumor growth and cancer recurrence. As many cancer patients suffer from intense pain, it would be unethical to withhold opioids based on the current evidence. In addition, it must be mentioned that opioid requirements are affected by multiple factors. More severe disease and postoperative complications are both associated with higher opioid requirements. However, both factors are also independent risk factors for cancer recurrence.

Prospective studies on the effect of regional anesthesia on cancer outcome have failed to show an advantage of regional analgesia over opioid analgesia. While using regional anesthesia to optimize patient comfort may be reasonable, there is no evidence that this approach improves patient outcome. Laboratory studies might hypothesize that higher plasma concentrations of lidocaine, which can be reached by perioperative intravenous infusion as a component of multimodal analgesia, might have an effect on outcome of cancer patients, but again large randomized controlled trials are missing.

There is evidence from meta-analyses that allogenic blood transfusions are associated with worse cancer outcomes. In general, a restrictive transfusion protocol should, therefore, be applied in cancer surgery.

Concerning other perioperatively administered drugs such as ketamine, α-2-agonists, and steroids, evidence is at best scarce regarding their impact on cancer outcome. Their use should be guided by the patient’s needs and not by the potential effect of these agents on cancer outcome.

**Table 1** summarizes the current evidence of the commonly used anesthetic agents on cancer progression.

**Table 1:** Summary of anesthetic effects on cancerous disease.

Drug or Method	Basic Research		Human Research		Conclusion, Recommendation
	*In vitro*	Animal	Retrospective	Prospective	
Volatile Anesthetics	↓		*	*	*
Propofol	Mostly ↑		*	*	*
	Breast Cancer ↔				
	Glioma ↓				
Volatile vs. Propofol	Propofol (↑)	Propofol ↑	Propofol (↑)	≈	Use propofol unless individually contraindicated
Opioids	↔	↔	(↓)	×	Use as clinically indicated
Regional Anesthesia	(↑)	─	↔	×	Use as clinically indicated
Local Anesthetics	Mostly ↑	Mostly ↑	─	─	Insufficient scientific data
Blood Transfusion		↓	↓	↓	Apply restrictive transfusion threshold
NSAIDs	↑	↑	↔	↔	Use as clinically indicated
Ketamine	─	↔	↔	×	Use as clinically indicated
α2-agonists	(↓)	↓	↔		Insufficient scientific data
Steroids	↔	↔	─	─	Insufficient scientific data

↓, unfavorable; *, see studies on volatile anesthetics vs. propofol; ↑, advantageous; (↑), advantageous in majority of studies; ≈, no difference between the two drugs; ↔, conflicting results; (↓), unfavorable in majority of studies; ─, no studies available; ×, no evidence of positive or negative treatment effect.

## Author Contributions

BB and MS performed the literature search and the manuscript preparation together. All authors contributed to the article and approved the submitted version.

## Conflict of Interest

The authors declare that the research was conducted in the absence of any commercial or financial relationships that could be construed as a potential conflict of interest.

## Publisher’s Note

All claims expressed in this article are solely those of the authors and do not necessarily represent those of their affiliated organizations, or those of the publisher, the editors and the reviewers. Any product that may be evaluated in this article, or claim that may be made by its manufacturer, is not guaranteed or endorsed by the publisher.
